# Risk Factors of Daunorubicine Induced Early Cardiotoxicity in Childhood Acute Lymphoblastic Leukemia: A Retrospective Study

**DOI:** 10.31557/APJCP.2021.22.5.1407

**Published:** 2021-05

**Authors:** Sunny Mariana Samosir, I Ketut Alit Utamayasa, Mia Ratwita Andarsini, Mahrus A Rahman, Teddy Ontoseno, Taufiq Hidayat, I Dewa Gede Ugrasena, Maria Christina Shanty Larasati, Andi Cahyadi

**Affiliations:** *Department of Child Health, Faculty of Medicine Universitas Airlangga/Dr Soetomo Academic General Hospital, Surabaya, Indonesia. *

**Keywords:** Anthracycline, cardiotoxicity, acute lymphoblastic leukemia

## Abstract

**Background:**

Daunorubicine, a type of anthracycline, is a drug commonly used in cancer chemotherapy that increases survival rate but consequently compromises with cardiovascular outcomes in some patients. Thus, preventing the early progression of cardiotoxicity is important to improve the treatment outcome in childhood acute lymhoblastic leukemia (ALL).

**Objective:**

The present study aimed to identify the risk factors in anthracycline-induced early cardiotoxicity in childhood ALL.

**Methods:**

This retrospective study was conducted by observing ALL-diagnosed children from 2014 to 2019 in Dr. Soetomo General Hospital. There were 49 patients who met the inclusion criteria and were treated with chemotherapy using Indonesian Childhood ALL Protocol 2013. Echocardiography was performed by pediatric cardiologists to compare before and at any given time after anthracycline therapy. Early cardiotoxicity was defined as a decline of left ventricle ejection fraction (LVEF) greater than 10% with a final LVEF < 53% during the first year of anthracycline administration. Risk factors such as sex, age, risk stratification group, and cumulative dose were identified by using multiple logistic regression. Diagnostic performance of cumulative anthracycline dose was evaluated by receiver operating characteristic (ROC) curve.

**Results:**

Early anthracycline-induced cardiotoxicity was observed in 5 out of 49 patients. The median cumulative dose of anthracycline was 143.69±72.68 mg/m^2^. Thirty-three patients experienced a decreasing LVEF. The factors associated with early cardiomyopathy were age of ≥ 4 years (PR= 1.128; 95% CI: 1.015-1.254; p= 0.001), high risk group (PR= 1.135; 95% CI: 1.016-1.269; p= 0.001), and cumulative dose of ≥120 mg / m^2^ (CI= 1.161; 95% CI:1.019-1.332).

**Conclusion:**

Age of ≥ 4 years, risk group, and cumulative dose of ≥120 mg/m^2^ are significant risk factors for early cardiomyopathy in childhood ALL.

## Introduction

Anthracycline group (e.g.daunorubicine, doxorubicin, and epirubicin) is still the mainstay of first-line chemotherapy for acute lymphoblastic leukemia (ALL) in children (Tripaydonis et al., 2019). As an antineoplastic agent, the mechanism of action in Anthracycline drugs include inhibition of deoxyribo nucleic acid DNA replication and ribo nucleic acid RNA transcription, particularly in rapidly dividing cells (i.e. cancer cells) and cardiomyocytes (Loar et al., 2018). 

Cardiotoxicity, as one of the side effects on the administration of anthracycline chemotherapy, has been widely reported (Krischer et al., 1997; Cardinale et al., 2015; Agha et al., 2016; Bansal et al., 2017). This alarming high incidence of cardiotoxicity can lead to failure of cancer therapy, therefore, this should be further investigated (Lipshultz et al., 2012). 

There are three different types of cardiotoxicities according to the time of onset, recognized as “acute”, occurring after a single course of anthracyclines or two weeks onset; “early”, developing within a year and being the most frequent form of cardiotoxicity; and “late-onset chronic”, occurring after the end of chemotherapy (Cardinale et al., 2015). The specific mechanism of cardiomyocyte injury from anthracyclines remains unclear. Most evidence implicates both enzymatic mitochondrial chain generation of free radicals and other nonenzymatic formations of anthracycline–iron complexes. The preferential involvement of the myocardium is not completely understood. Since mitochondria are one of the key mediators of anthracycline-induced cardiotoxicity, their abundance in cardiomyocytes could make them more vulnerable to injury. 

Different studies about cardiotoxicity in childhood ALL have portrayed various results. However, data on early cardiotoxicity risk factors in ALL children treated with anthracycline chemotherapy group have not been widely reported (Sadurska, 2015; Agha et al., 2016; Chellapandian et al., 2019). This study aimed to understand the risk factors of early cardiotoxicity in childhood ALL to reduce morbidity and mortality.

## Materials and Methods

*Study Design*

The present retrospective study was conducted on the medical chart of children with ALL in the Pediatric Ward of Dr. Soetomo General Hospital, Surabaya, Indonesia. From January 2014 to April 2019, 495 new children aged under 18 years were diagnosed with ALL based on bone marrow aspiration. Patients that were already treated with daunorubicine according to Indonesian ALL 2013 Protocol and had clear echocardiography data before and at any given time after daunorubicine administration were also included in this study. Patients with an incomplete medical record, critical congenital heart defect, and LVEF < 53% before chemotherapy were excluded. A total of 49 children met the inclusion criteria. Identification of risk factors, for instance, age, sex, risk group, and daunorubicine cumulative dose were obtained from medical records. 

*Assessment of risk factors*

Age - was classified into two groups. Group of children < 4 and ≥ 4 years old. Age was determined based on birth date and the day of the second echocardiography examination performed. Data on child sex were obtained from the medical record.

Stratification risk - was classified using Indonesian ALL 2013 Protocol stratification risk, consisting of high risk group (age < 1 year or > 10 years old, white blood cell level on diagnosis > 50,000/mm^3^, mediastinum mass > 2/3 thoracic diameter, blast cell > 5μm in cerebral spinal fluid) and standard risk group (age 1-10 years old and the initial white blood cell count < 50.000/mm^3^). The standard and high risk groups received a different dose of intravenous daunorubicine. The standard-risk group received twice administration of 30mg/m^2^ body surface area (BSA) daunorubicine and the high-risk group received four times of 30mg/m^2^ BSA daunorubicine during the induction phase and twice of 30mg/m^2^ BSA daunorubicin during the intensification phase. All infusion was given through 8 hours of intravenous drip diluted in 250ml NaCl 0.9%.

The cumulative dose - was calculated based on the total dose received between echocardiography divided with the patient’s BSA.

*Assessment of early cardiotoxicity*

Early cardiotoxicity was determined by the occurrence of decreasing LVEF > 10% or below 50% from the baseline within the first year of daunorubicine chemotherapy.

Cardiotoxicity was established based on the LVEF measured by a pediatric cardiologist through the M-mode view using The Logiq P6 Ultrasound System from General Electric (GE) Healthcare. 

*Statistical analysis*

Statistical measures (mean, standard deviation, and frequency distribution table) were arrayed as a descriptive analysis to reveal samples’ characteristics. Calculation on cut off dose was analyzed using receiver operating characteristic curve. Bivariate and multivariate analysis using multiple logistic regression were performed. Result was considered significant if p < .05. Data analysis was conducted by using IBM SPSS ver. 22.0.

*Ethical clearance*

The study protocol was assented by the Research and Development Deputy of Dr. Soetomo Hospital. The ethical clearance was declared by the Ethical Committee of Dr. Soetomo Hospital (No. 1156/KEPK/V/2019).

## Results

*Characteristic of patients*

Table 1 shows the characteristics of the subjects involved in this study. There were 18 females (36.7%) and 31 males (63.3%) with a mean age of 9.18 (range 2-16 years old). Among all subjects, there were 5 children aged less than 4 years old (10.2%) and the rest of them were ≥ 4 years (89.8%). In this study, the high-risk group dominated with a total of 42 children (85.7%). About 5 children experienced early cardiotoxicity (10.2%). 

*Identifying Risk Factors of Daunorubicine-induced Early Cardiotoxicity*

Bivariate analysis revealed that age of ≥ 4 years, risk group, cumulative dose of 120mg/m2 were potentially associated with early cardiotoxicity (all p <.05, see Table 1). The potential risk factors were then introduced to the multivariate analysis and multiple regression logistic. 

The risk factors of Daunorubicine-induced early cardiotoxicity are summarized in Table 2. The risk of children aged ≥4 years old was 1.128 times higher than those aged <4 years old (prevalence ratio, PR 1.128; 95% CI: 1.015 – 1.254; p < .001). The high-risk group had 1.135 times more risk of cardiotoxicity than the standard-risk group (PR 1.135;95%CI: 1.016 – 1.269; p < .001). While the risk of ALL children with cumulative dose above 120mg/m2 experiencing early cardiotoxicity was 1.161 times higher than those with lesser dose (PR 1.161; 95%CI: 1.019 – 1.324; p 0.001). 

*Cut off dose estimation*

**Figure 1:**
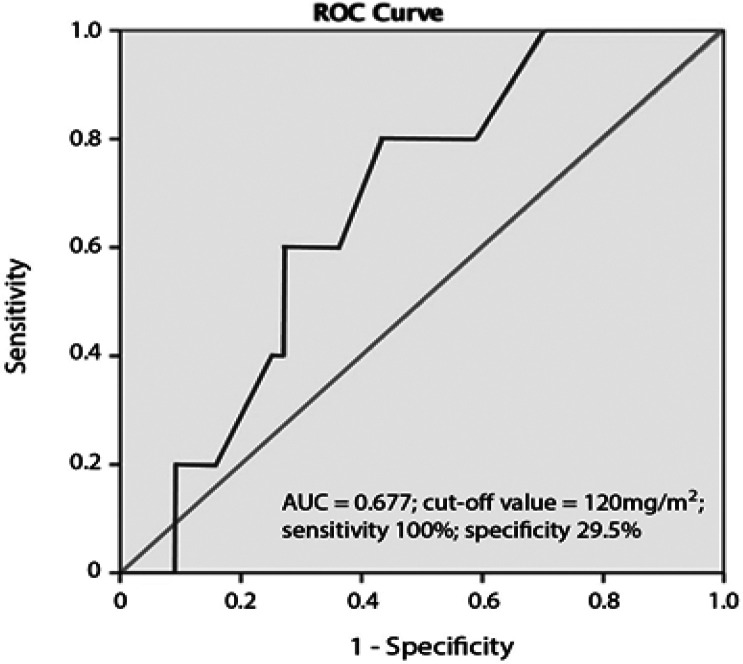
ROC Analysis of Daunorubicine Cumulative Dose for Predicting Early Cardiotoxicity in Childhood ALL in Dr. Soetomo General Hospital

The diagnostic value of the cumulative anthracycline dose was further explored using ROC curve. The area under the ROC for cumulative dose was 0.677. The sensitivity and specificity values were 100 and 29.5%, respectively, at a cut-off value of cumulative dose >120 mg/m2. (see Figure 1).

**Table 1:** Demographic and Clinicopathological Characteristics of ALL-Diagnosed Children from 2014 to 2019 in Dr. Soetomo General Hospital, Indonesia

Characteristics	Category	Early cardiotoxicity		P
		Yes	No	
Child Sex	Male	2 (6.5%)	29 (93.5%)	0.152
	Female	3 (16.7%)	15 (83.3%)	
Age	<4 years	0 (0.0%)	5 (100.0%)	0.001*
	≥4 years	5 (11.4%)	39 (88.6%)	
Risk group	Standard risk	0 (0.0%)	7 (100.0%)	0.001*
	High risk	5 (11.9%)	37 (88.1%)	
BSA	mean ± SD (range)	1.07 ± 0.20	0.97 ± 0.29	0.483
		(0.80 – 1.28)	(0.50 – 1.63)	
Cummulative dose	mean ± SD (range)	179.04 ± 39.78	138.45 ± 85.52	0.087
		(135.6 – 228.0)	(16.1 – 417.0)	
Cummulative dose/m^2^ BSA	mean ± SD (range)	170.76 ± 39.24	140.61 ± 72.68	0.175
		(120.00 – 225.65)	(24.96 – 386.49)	
	<120 mg/m^2^	0 (0.0%)	13 (100.0%)	0. 001*
	≥120 mg/m^2^	5 (13.9%)	31 (86.1%)	

Data are presented as n (%) or mean (SD). BSA, body surface area; SD, standard deviation

**Table 2:** Prevalence Risk of Daunorubicine Early Induced Cardiotoxicity in Dr. Soetomo General Hospital, Indonesia

Parameters	P value	Prevalence Risk (95% C.I)
Children age		
< 4 years	Referent	
> 4 years	<0.001	1.128 (1.015 – 1.254)
Risk		
Standard Risk	Referent	
High Risk	<0.001	1.135 ( 1.016 – 1.269)
Cumulative Dose		
< 120 mg/m^2^	Referent	
> 120 mg/m^2^	0.001	1.161 (1.019 – 1.324 )

## Discussion

Anthracyclines are the most commonly used antineoplastic medication to treat hematological and solid tumors. Hence, the growing rate of cancer survival, the clinical significance of anthracycline cardiotoxicity, is an emerging medical issue (Nebigil and Desaubry, 2018). The precise mechanism of anthracycline-induced cardiotoxicity in ALL is still not well understood (Bansal et al., 2017). These cardiotoxic effects are categorized as acute, early, and late depending on the times since anthracycline administration (Cardinale et al., 2015). Most studies regarding anthracycline cardiotoxicity were designed for the assessment of late-onset cardiotoxicity. Anthracycline-induced cardiotoxicity within the first year of the treatment was investigated in a few studies (Baysal et al, 2010; Kocabas et al., 2014; Cardinale et al., 2015).

We used the echocardiographic measurement of LVEF as the endpoint in this study. Five children presented with cardiotoxicity presumably caused by daunorubicin, one of the derivatives in the anthracycline group. A study with larger population consisting of 6,493 children reported that about 1-2% of the children treated on frontline cancer protocols had symptomatic cardiac dysfunction within a year after treatment. This alarmingly higher incidence of cardiotoxicity could be attempted due to the different terminology used. Krischer et al., (1997) stated that cardiotoxicity was defined as congestive heart failure, abnormal measurements of cardiac function that prompted discontinuation of therapy, or sudden death from presumed cardiac causes. While in the present study, cardiotoxicity was defined as > 10% decreasing LVEF from baseline or less than 50%, even without clinical manifestation of heart failure.

In accordance with the present study, it is shown that age of ≥4 years old was a significant risk factor for early cardiotoxicity in childhood ALL. Left ventricle volume, mass, and function vary over ages in healthy individuals. The downward trend in ejection fraction (EF) values may be part of a reasonable aging process. Studies on normal values of echocardiography in healthy individuals using magnetic resonance imaging (MRI) showed a rapid EF decline in adolescence compared to adulthood (Cain et al., 2009). In contrast, a cohort study by Van der Pal (2011) reported that younger age at diagnosis had a clinically important role in the decline of subclinical heart function evaluated with left ventricular shortening fraction (LVSF) parameter. Pharmacokinetic and pharmacodynamic studies in children with cancer showed significantly lower drug clearance in the less 3 years old age group. Such finding supported the claim that younger age becomes one of the risk factors for cardiotoxicity in cancer patients (Krischke et al., 2016). However, the result in this current study might be affected by the selection bias due to the larger proportion of samples namely age of ≥4 years old.

This study depicts that gender is not a risk factor for anthracycline-induced cardiotoxicity. Similar results were also reported in Cuba from children with ALL who received anthracycline therapy (Otero et al., 2016). In contrast to the results obtained by the present study, a cohort study of 150 cancer survivors treated with anthracycline therapy for women had 3.2 times greater risk than men to have cardiac dysfunction based on angiography and electrocardiography examination (Meiners et al., 2018). Some literatures that support female sex as a risk factor for heart problems due to anthracycline exposure in childhood cancer presented differences in body composition as a contributing factor to sexual dysmorphism. Women had more body fat than men. Anthracycline is not distributed in fat tissue, whereas, higher concentrations can be found in other tissues such as the heart (Webster-Gandy et al, 2003; Meiners et al., 2018).

In our study, children with high-risk stratification were dominating (85.7%). All patients presented with early cardiotoxicity were from this group, thus, a significant correlation was observed. In Indonesia, there are only two categories in terms of risk stratification based on Indonesian Protocols of 2013 Childhood ALL chemotherapy. The higher risk group gets about three times higher dose than the standard risk group during chemotherapy cycle (Indonesian Pediatric Society, 2013). Wau et al., (2017) revealed a higher number of heart defect events observed in the high-risk group than the standard-risk group of childhood ALL. Conversely to the previous research, Gunawan et al., (2018) concluded that there was no significant correlation between the risk group and the incidence of cardiotoxicity in ALL children treated with an anthracycline. Here, the subjects in the standard-risk group were bigger than the other risk stratification. This difference could be existing due to the high mortality rate of high-risk survivors in Indonesia.

There are various protocols and numbers of risk stratifications in the world, which make the accumulated dose parameter more common to use (Indonesian Pediatric Society, 2013; Terwiliger et al., 2017; Ariffin et al., 2020). Prior studies in anthracycline-induced cardiotoxicity documented cardiologic abnormalities associated positively with a higher dose of anthracycline drugs given (Lipshultz et al., 2014; Bansal et al., 2017; Bansal et al., 2019; Armenian and Bhatia, 2020). Of the 49 children in this study, 5 children experienced a decrease of >10% and <53% in LVEF, met cardiotoxicity criteria according to the American Society of Echocardiography and the European Association of Cardiovascular Imaging (Perez et al., 2019). In the present study, the multiple regression of cumulative dose and early cardiotoxicity showed no correlation. However, when being analyzed using a certain cut off values, there was an increasing risk of cardiomyopathy up to 1.161 times in pediatric ALL patients who had received an accumulated anthracycline dose of 120 mg/m^2^ BSA. The determination of cut-off values was based on ROC analysis. A cumulative dose of 120 mg/m^2^ provided 100% sensitivity and a specificity of 29.5%, of which the number was good for screening the risk of cardiotoxicity. The certain cut off point was based on previous study in Dr.Soetomo General Hospital, where a cumulative dose of 120 mg/m^2^ began to impair left ventricular diastolic function (Rahman, 2016). Inversely, a multicenter study in the Netherlands reported that no heart damage was obtained based on physical examination, electrocardiography, and echocardiography in ALL children who received daunorubicin chemotherapy at a lower dose of 100 mg/m^2^ divided into 4 doses administration each week (Rammeloo et al., 2000).

The authors got different results in this study in which none of the patients were recorded as having clinical heart failure during the evaluation echocardiography. The size of the observation period could also be the reason why the present study did not find patients with clinical heart failure. Van Dalen et al., (2006) stated that cancer survivor who got anthracycline with observation range of 0.01-28.4 years mentioning a value of 300mg/m^2^ had a relative risk (RR) = 8 independent of clinical heart failure. While another cohort study of cancer survivors who got anthracycline with an observation range of 0.01 – 28.4 years mentioning a value of 300 mg/m^2^ as an independent variable of clinical heart failure, with relative risk (RR) = 8.

The term “no safe dose in the administration of anthracycline” needs to be properly observed. Previous research had shown that results were varied between the dosage of anthracycline and cardiotoxicity due to the variety of parameters used, the time range of observations, and the methodology used. Cumulative exposure to anthracycline in various current chemotherapy protocols varied greatly between 75 – 450 mg/m^2^. However, there had been no plans for dose reduction in the near term up to now. Some of the proposed strategies for reducing the risk of cardiomyopathy included reducing other cardiotoxic drugs, the selection of types of encapsulated liposomal doxorubicin in adult patients, long-term infusions, as well as protective cardioprotective agents such as dexrazoxane (Armenian and Bhatia, 2020).

The limitation of this study is the use of secondary data only from medical records without the same evaluation period. The study focused solely on evaluating LVEF values regardless of other cardiac hemodynamic measurements. The sample limitation is also a shortcoming in this study. Nonetheless, the authors try to overcome it by doing bootstraps during statistical analysis.

In conclusions, Age of ≥ 4 years, high-risk group, and cumulative dose exceeding 120 mg/m^2^ are the risk factors for the incidence of early cardiotoxicity in pediatric ALL patients who get daunorubicin chemotherapy. We recommend that a cumulative dose > 120mg/m2 might be a valuable indicator for the prediction of early cardiotoxicity in childhood ALL treated with daunorubicin.

## Author Contribution Statement

The author’s contributions are described as the following: Sunny Mariana Samosir, I Ketut Alit Utamayasa, Mia Ratwita Andarsini, Teddy Ontoseno, I Dewa Gede Ugrasena, Maria C. Shanty Larasati, and Andi Cahyadi designed and conducted the initial analysis. Utamayasa, Mahrus Rahman, and Taufiq Hidayat performed the echocardiography. Samosir collected the data, conducted further analysis, and did the manuscript writing. Finally, all authors had read and approved the final version of this manuscript.
